# Socioeconomic Drivers of PM_2.5_ in the Accumulation Phase of Air Pollution Episodes in the Yangtze River Delta of China

**DOI:** 10.3390/ijerph13100928

**Published:** 2016-09-22

**Authors:** Cai-Rong Lou, Hong-Yu Liu, Yu-Feng Li, Yu-Ling Li

**Affiliations:** 1 Key Laboratory of Virtual Geographic Environment, Nanjing Normal University, Ministry of Education, Nanjing 210023, China; loucairong@126.com (C.-R.L.); pandalee_0826@163.com (Y.-F.L.); ndslyl@163.com (Y.-L.L.); 2 College of Geographic Sciences, Nantong University, Nantong 226007, China; 3 State Key Laboratory Cultivation Base of Geographical Environment Evolution (Jiangsu Province), Jiangsu Center for Collaborative Innovation in Geographical Information Resource Development and Application, College of Geographical Science, Nanjing Normal University, Nanjing 210023, China

**Keywords:** PM_2.5_, pollution episode, socioeconomic factor, geographical detector, Yangtze River Delta

## Abstract

Recent studies in PM_2.5_ sources show that anthropogenic emissions are the main contributors to haze pollution. Due to their essential roles in establishing policies for improving air quality, socioeconomic drivers of PM_2.5_ levels have attracted increasing attention. Unlike previous studies focusing on the annual PM_2.5_ concentration (*C_year_*), this paper focuses on the accumulation phase of PM_2.5_ during the pollution episode (PMAE) in the Yangtze River Delta in China. This paper mainly explores the spatial variations of PMAE and its links to the socioeconomic factors using a geographical detector and simple linear regression. The results indicated that PM_2.5_ was more likely to accumulate in more developed cities, such as Nanjing and Shanghai. Compared with *C_year_*, PMAE was more sensitive to socioeconomic impacts. Among the twelve indicators chosen for this study, population density was an especially critical factor that could affect the accumulation of PM_2.5_ dramatically and accounted for the regional difference. A 1% increase in population density could cause a 0.167% rise in the maximal increment and a 0.214% rise in the daily increase rate of PM_2.5_. Additionally, industry, energy consumption, and vehicles were also significantly associated with PM_2.5_ accumulation. These conclusions could serve to remediate the severe PM_2.5_ pollution in China.

## 1. Introduction

In past decades, with the rapid development of industrialization and urbanization, the problem of air pollution has become increasingly severe around the world. In particular, fine particulate matter (PM_2.5_), a type of pollutant, has been verified to be unhealthy for humans and the living environment, as it can cause lung cancer, respiratory and cardiovascular diseases, affect transportation, as well as increase mortality [1,2,3,4]. Recently, PM_2.5_ pollution has become the greatest environmental problem in China and is therefore attracting growing public concern. Since 2013, heavy PM_2.5_ pollution events have often occurred in many Chinese cities, as observed via monitoring-site data [5] and satellite imagery [6,7]. For instance, in the Yangtze River Delta (YRD), a developed region in China, Shanghai witnessed a maximum hourly PM_2.5_ concentration of 602 μg/m^3^ [8] in December of 2013. In addition, Nanjing experienced a daily PM_2.5_ concentration (*C_day_*, the average concentration of 24 h in a day) of 369 μg/m^3^, which is far above 75 μg/m^3^ (the 24-h standard of Chinese Ambient Air Quality Standards, CAAQS, GB3095-2012). In 2014, 86 million people in some cities of the YRD were exposed to more than 100 pollution days (*C_day_* ≥ 75 μg/m^3^), so there is an urgent need to formulate effective policies to improve the severely polluted air environment in China.

A considerable body of literature describes the enormous efforts dedicated to understanding PM_2.5_. It is mainly focused on the chemical composition [9,10,11] and source apportionment [10,12,13], spatiotemporal variations of ambient concentration [14,15,16] and various influencing factors (such as human emissions, synoptic conditions, topography, and vegetation, etc.) [8,17,18,19]. Among them, the analyses regarding the influence of socioeconomic factors (SEFs), such as population, population density, and vehicles, etc., on PM_2.5_ pollution can better assist environmental policy-makers. Several articles have identified industrial activities, fuel combustion, biomass burning, and vehicles as major contributors in the YRD [13,20]. Wang et al. [21] explored PM_2.5_ contributors in Shanghai from 2011 to 2013 and identified three kinds of typical PM_2.5_ episodes, caused by biomass burning, suspended dust, and fireworks. They suggested that gas precursors from human activities and their secondary formation dominated PM_2.5_ pollution. Ye et al. [17] also mentioned some cultural customs, like fireworks and the burning of incense sticks during the Chinese New Year period. They indicated these were the less-obvious factors resulting in PM_2.5_ pollution episodes. Furthermore, using satellite detection Zhang et al. [22] determined that the annual PM_2.5_ emissions from open straw burning were 1.036 metric tons in China during 1997–2013. These studies have all reported strong links between PM_2.5_ pollution and human behavior, although most of them mainly investigated a certain source or a specific period of time. For decision-makers to formulate an environmental control target, it is important to fully understand the impact of socioeconomic development on air pollution from a statistical perspective and recognize the force of local socioeconomic drivers.

However, studies relating to the inextricable links between PM_2.5_ and SEFs are still scarce. It is only in recent years that several articles have tried to apply quantitative analysis methods to this problem. One reason for this could be that high PM_2.5_ concentrations occur most frequently in developing countries, where pollution data was unavailable. According to the latest studies, several articles have argued that some SEFs are closely related to ambient PM_2.5_ concentration. For instance, in 1999, Romero et al. [23] explored the relationship between rapid urban growth and air pollution in Santiago. He suggested that the highest population concentration mainly occurred in the urban area and determined industries and vehicles were the main contributors to smog. Using Pearson Correlation analysis, Han et al. [6] showed the annual PM_2.5_ concentration (*C_year_*, the mean concentration of all days in a year) in urban areas was positively related to urban population and an urban secondary industrial fraction at the Chinese prefectures level. Hao and Liu [5] employed the Spatial Lag Model and Spatial Error Model to examine the relationship between urban air quality and the socioeconomic development in China, suggesting that secondary industry and vehicle population positively influenced *C_year_*. In addition, Wang and Fang [16] discussed the influence of SEFs on PM_2.5_ in the Bohai Rim urban agglomeration using Geographically Weighted Regression (GWR) and further explored their quantitative relationships. They concluded that in some cities of that region, an increase of 10,000 yuan in the GDP per capita would reduce PM_2.5_ of 1.18 μg/m^3^, while a 1% increase in urbanization rate and an increment of 10,000 in the working population could raise PM_2.5_ by 1.25 μg/m^3^ and 0.09 μg/m^3^, respectively. Generally speaking, these studies addressing the statistical links between SEFs and PM_2.5_ have mainly focused on *C_year_*, with discussions on the national scale, as well as urban scale. However, *C_year_* is influenced by many factors, such as human emissions, synoptic conditions, surface terrain and vegetation, etc. The difference of these factors may be enormous at the national scale, but not significantly different within a city. Thus, it is considered that *C_year_* may not be appropriate to reflect the influence of SEFs under the interference from other factors. To accurately evaluate the impact of SEFs on PM_2.5_, it is necessary to minimize this disturbance as far as possible.

For the aforementioned reasons, we selected the YRD as the sample area, focusing only on the relationship of SEFs with PM_2.5_ in the accumulation stage during in the pollution episodes, called PMAE in this study. Two main aspects were considered. Firstly, each pollution episode was split into an accumulation stage and a diminishing stage, based on the peak concentration. Unlike the diminishing process which is controlled by winds and precipitation [24], the accumulation stage generally occurs under stagnant weather conditions [13,25]. Further, cities in the YRD had similar synoptic conditions, as a pollution episode typically only lasted for a few days. Thus, our focus on PMAE could greatly reduce the interference from the different weather conditions to some extent. Secondly, although the YRD was often regarded as a whole in numerous studies, the socioeconomic development here as well as the atmospheric pollution, did differ among cities in the region. For example, in 2014, Shanghai had a population of 24 million, more than 34 times that of Zhoushan. This difference brings about many distinctions with regard to the other SEFs between the two cities, like the total energy consumption and the number of vehicles in Shanghai, which were found to be 98 and 27 times higher, respectively, than those in Zhoushan. Given the minimal difference in the synoptic environmental conditions in the YRD, the spatial diversity of PMAE may be mainly dominated by socioeconomic drivers. However, to our knowledge, existing studies show a lack of concern about the relationship between PMAE and socioeconomic factors, especially at a regional scale.

Regarding the analysis methods, various models, such as the traditional Ordinary Least Square model, Land Use Regression [26,27,28], GWR [16], Panel Data model [29], Spatial Lag model and Spatial Error model [5], were adapted to quantify the relationship between various SEFs and PM_2.5_. However, due to their collinearity, many SEFs were removed in the abovementioned models. To compare the influence of each indicator on PM_2.5_ at the regional level, we used a geographical detector to explore the spatial correlations between PMAE and SEFs.

Overall, we first selected 10 typical pollution episodes from 2014 and averaged them to obtain the characteristics of PMAE in the YRD. Then, using the geographical detector and a linear regression model, the following goals were pursued, as presented in the subsequent sections: to explore the relationship between PMAE and SEFs, as well as to identify the influence of the socioeconomic indicators driving the accumulation of PM_2.5_. Some of the main findings are discussed at the end of this paper. All of these should provide policy-makers with an insight into PM_2.5_ accumulation during pollution episodes in order to formulate appropriate air quality regulations.

## 2. Methods

### 2.1. Sample Area and Cities

**Figure 1:**
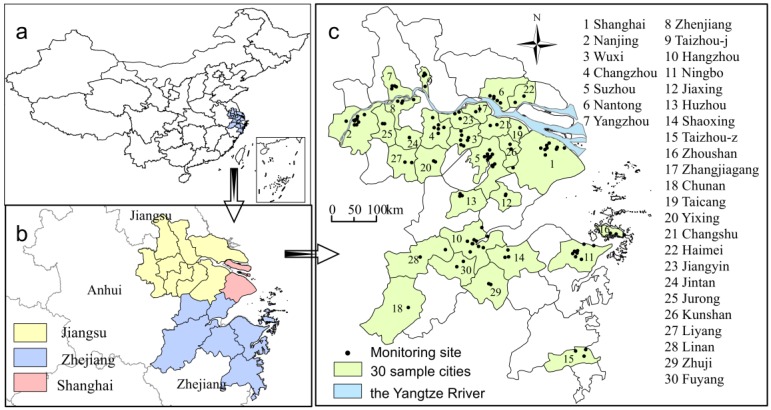
The location of the YRD in China (**a**); the three parts of the YRD in this study; (**b**); and the sample cities and the location of 120 observation sites in the YRD (**c**).

In our study, the YRD consists of Shanghai, the southern part of Jiangsu Province and the northeastern part of Zhejiang Province, including 16 cities (Figure 1a,b). Being a developed area, although this region covers only 1.1% of China in terms of area, it accounts for 7.47% of its total population (according to the data of 16 cities in 2014). Due to the dense urban clusters and the increasing coal consumption in recent years, the region has frequently suffered from severe PM_2.5_ pollution events. On account of the negative effects on human health, PM_2.5_ concentration has been automatically monitored in many cities in China since 2013. Considering that most of the monitoring sites are located in the urban built-up area. We chose the urban area of 16 prefecture-level cities and 14 counties for this study, including at least one air-monitoring site, as shown in Figure 1c.

### 2.2. PM_2.5_ Data

#### 2.2.1. Ambient PM_2.5_ Concentration

Currently, the PM_2.5_ concentration data are updated every hour on the air quality publishing platform of the National Environmental Monitoring Centre in China. This paper derived hourly PM_2.5_ concentrations from 120 monitoring sites in the 30 sample cities in the YRD from 1 January to 31 December in 2014. According to the requirements for the validity of the concentration of air pollutants released in GB3095-2012, firstly, we deleted the values ≤0 and the abnormal concentrations in the raw data. Secondly, a daily concentration (*C_day_*) was calculated by averaging the value for 24 h from 0:00 to 23:00. If the hourly data were missing for more than 4 h on any day, the *C_day_* was considered invalid and excluded. At last, for each monitoring site, the mean of all daily concentrations was seen as its *C_year_*, and then the average *C_year_* of all sites in a city represented its urban pollution level.

#### 2.2.2. Pollution Day and PM_2.5_ Episodes

**Figure 2:**
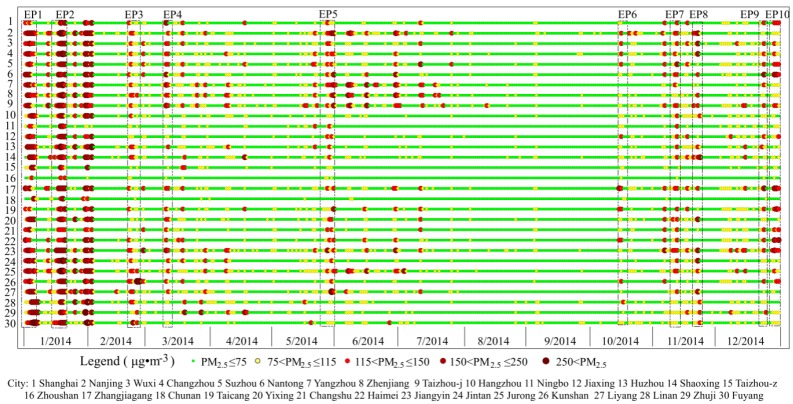
Daily PM_2.5_ concentrations and ten pollution episodes selected in 2014.

To explore the characteristic of pollution episodes clearly, we defined a pollution day as a day with *C_day_* ≥ 75 μg/m^3^, and a pollution episode as the pollution period with more than two consecutive pollution days. To reduce abnormality and contingency, ten pollution episodes were carefully picked out to average. The principles of selecting PMAE are as follows: (1) there is no daily data missing during a pollution episode; (2) there is no precipitation, and the wind conditions had a value of less than 3 on the Beaufort scale; and (3) the pollution episodes are distributed in different seasons. Although the *C_day_* in part cities perhaps have not reached the pollution level as defined above, the same PM_2.5_ changing period was chosen simultaneously in all cities for comparing. As a result, ten pollution events, EP1-EP10 (during in the time of 1/1–1/6, 1/15–1/21, 2/19–2/23, 3/8–3/11, 5/26–5/30, 10/13–10/17, 11/9–11/13, 11/16–11/21, 12/20–12/26, and 12/27–12/31, respectively) were adapted and selected for this paper (Figure 2) at last. Due to frequent rainfall, no episode was eligible from June to September.

### 2.3. Method

#### 2.3.1. PM_2.5_ Episode Indexes

To exhibit the characteristic of PMAE, We defined three episode indexes, *MI_ep_*, *DI_ep_*, and *AD_ep_*, which are written as follows:

(1) $$MI_{ep}=\frac{1}{n}\sum_{i=0}^{n}\left(C_{\left(max,i\right)}-C_{\left(0,i\right)}\right)$$

(2) $$DI_{ep}=\frac{1}{n}\sum_{i=0}^{n}\frac{MI_{ep,i}}{d_{i}}$$

(3) $$AD_{ep}=W_{1}MI_{ep}+W_{2}DI_{ep}$$

where, *MI_ep_*, *DI_ep_*, and *AD_ep_* refer to the maximum increment, the daily increase rate and the accumulation degree of PM_2.5_ during a pollution episode, respectively. *i* = 1, 2, 3……*n* (*n* = 10). *C*_(*0, i)*_ and *C*_(*max, i)*_ refer to the beginning and the peaking concentration of PM_2.5_ in EP*i*, respectively. *d_i_* is the days of duration before PM_2.5_ concentration reaching the peak in EP*i*. In Equation (3), *W*_1_ and *W*_2_ represent the weight of *MI_ep_* and *DI_ep_*, respectively. In this study, considering *MI_ep_* and *DI_ep_* were significantly positively related to each other (*R*^2^ = 0.79), we defined *W*_1_ = *W*_2_ = 0.5. When the Equation (3) was applied, *MI_ep_* and *DI_ep_* were normalized to the region of (0, 1) first. The higher value of *AD_ep_* indicates PM_2.5_ in that city is easier to accumulate.

#### 2.3.2. Geographical Detector Model

The geographical detector proposed by Wang et al. [30] is a novel and suitable spatial analysis method to detect the influential force on certain geographic and environmental phenomena, which has been applied in many fields in recent years [31,32,33]. We used factor detector, one module of the geographical detector, to determinate the impact of socioeconomic indicators on PMAE in our study. Let one SEF be D, which is categorized into several sub-region *D_i_* (*i* = 1, 2, 3……*m, m* is the number of sub-region, and m = 5 in this study), and let an episode index (i.e., *MI_ep_*, *DI_ep_*, or *AD_ep_*) be *H*, then the determinant power of factor *D* to *H* (*PD_D,H_*) could be expressed as follows [30,31,33]:

(4) $$PD_{ep}=1-\frac{1}{n\sigma^{2}}\sum_{i=1}^{m}n_{D,i}\sigma_{D,i}^{2}$$

where, *n* and *n_D,i_* are the number of samples in the total study area and in the divisional *D_i_*, respectively; *σ*^2^ and $\sigma_{D,i}^{2}$ refer the variations of *H* in the total study area and in the divisional *D_i_*. The higher value of *PD_D,H_*, which is between 0 and 1, indicates the impact of the factor D on PM_2.5_ is stronger.

#### 2.3.3. Factor Detector Indicators

**Figure 3:**
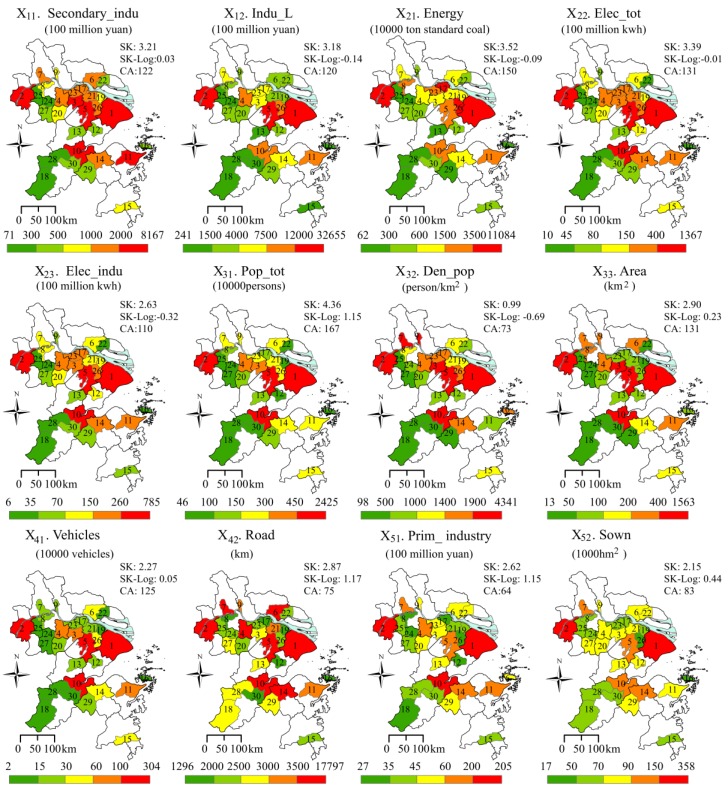
Spatial distributions of socio-economic factors. (SK is the skewness of the SEFs; SK-Log refers the skewness of SEFs used in a logarithmic form. CA is the coefficient of variation, which is calculated by the equation “CA = (Mean/Standard Deviations) × 100”. Each city was numbered as in Figure 1. The blue color is the Yangtze River.)

The analysis of PM_2.5_ sources was a focus in previous articles. Firstly, pursuant to several papers, industry, coal combustion, private vehicles, gas combustion, iron and steel manufacturing, and biomass burning were regarded as the main sources generating fine particulate matter in the YRD [13,34]. Secondly, some studies have suggested that city size impacts air quality. For instance, Stone [35] and Martins [36] suggested that urban sprawl led to changes in population, energy consumption and air emissions, which would finally result in a worsening of air quality. Thirdly, population density was considered an influencing factor, but it is unclear whether the population density plays a positive or negative role with regard to PM_2.5_. On the one hand, a higher density in a large city is helpful for pollution-concentrated disposal, which will improve environment [35]. On the other hand, a higher density of population would cause more energy consumption and more emissions. As reported in many studies, the ambient PM_2.5_ concentration is higher in an urban area than in a rural region [6,37], indicating that higher population density can aggravate PM_2.5_ pollution. To further explore the effects of social and economic factors on PMAE, we chose five pollution sources, including 12 indicators, as detector factors (Table 1). Considering the monitoring sites were located in the urban built-up district, each variable in this paper was considered at an urban area scale to match the PM_2.5_ data. All the SEFs data, which was captured as annual values, came from the Statistical Yearbook (2014) [38,39,40] or the National Economic and Social Development Statistics Bulletins of 30 cities (http://www.stats.gov.cn/tjgz/wzlj/dftjwz/). Figure 3 shows the spatial distributions of the twelve SEFs. In the remainder of this paper, the abbreviations of the indicators as follows: X_11_, Sec_indu; X_12_, Indu_L; X_21_, Energy; X_22_, Elec_tot; X_23_, Elec_indu; X_31_, Pop_tot; X_32_, Den_pop; X_33_, Area; X_41_, Vehicles; X_42_, Road; X_51_, Prim_indu; X_52_, Sown.

#### 2.3.4. Univariate Linear Regression

It is noteworthy that a positive or negative correlation did not indicate causality completely. To express the force of each SEF on PMAE, we employed univariate linear regression to determinate the influence. Linear regression is a model used for data following a normal distribution. Due to the skewed distribution of many socioeconomic data (Figure 3), we select a log transformation to process the raw indicators. Compared with SK (the skewness of initial data), the values of SK-Log (the skewness of data in a logarithmic form), as shown in Figure 3, had a significant decrease from 2.68 ± 1.38 to 0.24 ± 0.93. Thus, a log transformation is considered to be a better effective method for processing the data and was selected in this study. The linear fitting formula is written as Equation (5):

(5) Ln(Y)=a+bLn(X)

where, *Y* is an episode index (*MI_ep_*, *DI_ep_*, or *AD_ep_*); *X* means the socioeconomic indicators in Table 1; a is constant, and b stands for the slope of the regression line. This paper used the slope b to express the elasticity of *Y* growth caused by per unit of *X* added.

## 3. Results

### 3.1. Characteristics of PM_2.5_ Pollution in the YRD

**Figure 4:**
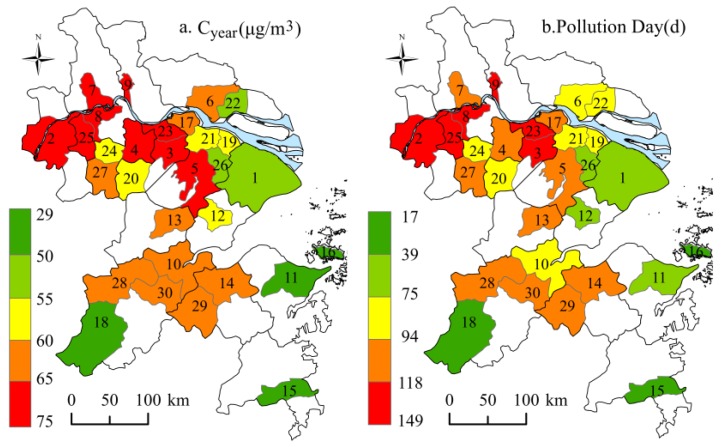
Spatial distribution of the annual PM_2.5_ concentrations and pollution days in sample cities in 2014. (Each city was numbered as Figure 1. The blue color is the Yangtze River.)

**Figure 5:**
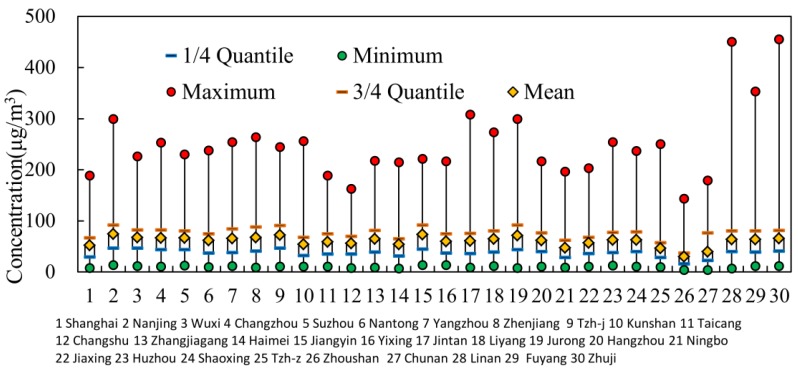
Statistics of daily PM_2.5_ concentrations in sample cities in 2014.

In 2014, the YRD experienced long-duration PM_2.5_ pollution, as shown in Figure 2. The regional *C_year_* was 62.57 μg/m^3^, which is higher than the national average (61 μg/m^3^) [16] and obviously exceeds the CAAQS 35 μg/m^3^ *C_year_* standard. Affected by various factors, the PM_2.5_ concentration was highest in winter (100.52 μg/m^3^), followed in spring (62.28 μg/m^3^) and in fall (52.49 μg/m^3^), and lowest in summer (49.61 μg/m^3^). Figure 4a,b show the spatial variation of *C_year_* and pollution days, respectively. Figure 4 indicates that the cities with higher *C_year_* were mainly located in the northwest of the region. For example, the top four cities, Nanjing, Taizhou, Jurong, and Jiangyin, which were located along the Yangtze River, had the higher *C_year_* of 74.63, 73.53, 73.17, and 73.17 μg/m^3^, respectively. Only one out of 30 sample cities, i.e., Zhoushan, witnessed a *C_year_* of less than 35 μg/m^3^. There were 16 cities with more than 100 pollution days. Figure 5 shows the statistical results of *C_day_*. In the highly-polluted days, the *C_day_* reached a peak of 454 μg/m^3^ in Zhuji, 449 μg/m^3^ in Lin’an, and 143–352 μg/m^3^ in other cities, far in excess of 75 μg/m^3^. Regarding spatial distribution, the value of *C_year_* and pollution days all decreased from the northwest to the southeast, which may be related to the coastal location.

### 3.2. Characteristics of PMAE

**Figure 6:**
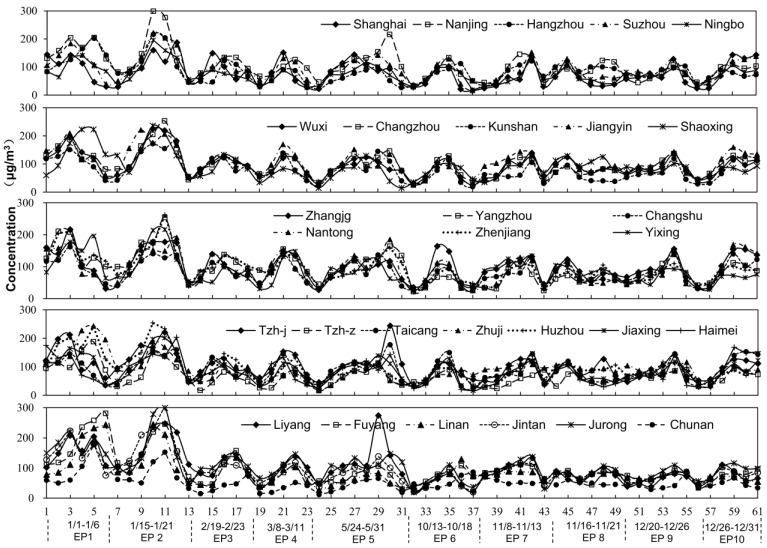
Ten PM_2.5_ episodes from the sample cities in the YRD.

To express the results intuitively, the 30 cities were divided into five groups based on their GDP in 2014. Ten selected pollution episodes from these cities have been shown in detail in Figure 6. Table 2 showed the statistics of pollution indicators during the ten episodes in the YRD. The results indicated that severe episodes mainly occurred in January. In particular, during EP1 and EP2, the *C_day_* peaked at 143–307 μg/m^3^ in the sample cities. During the ten selected episodes, the maximum *of MI_ep_* and *DI_ep_* reached 217 μg/m^3^ and 55.42 μg/(m^3^·d) (observed in Nanjing). Notably, a peak was noticed in May, during EP5, for instance, when the maximum *C_day_* reached 84–273 μg/m^3^ in all the cities.

**Figure 7:**
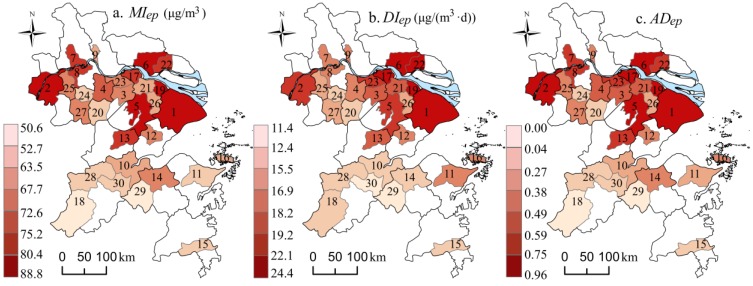
Spatial patterns of PM_2.5_ episode indexes in the YRD. (Each city was numbered as Figure 1. The blue color is the Yangtze River.)

From Figure 6, it can be seen that every PM_2.5_ pollution episode in each city in the YRD showed a similar, but not identical, tendency. This means the upward and downward trends in each city were similar, while their peak concentrations and the corresponding time of appearance were different. Unlike the spatial variations of *C_year_* (decreasing from northwest to southeast), the high values of *MI_ep_*, *DI_ep_*, and *AD_ep_* (obtained by averaging ten episodes) were concentrated on both sides of the Yangtze River, in addition to being characterized by a downward trend from north to south (Figure 7a–c). For instance, in Shanghai, PM_2.5_ reached the peak faster, during six out of ten episodes, than in the other cities. Although Shanghai experienced a relatively lower *C_year_* at 51.97 μg/m^3^, it saw the highest *MI_ep_* at 88.73 μg/m^3^, *DI_ep_* at 23.78 μg/(m^3^·d) and *AD_ep_* at 0.97 among the 30 cities. That is probably because of the dense population and vast industrial activities, which would cause more air emissions compared to the other cities. Another example, Nanjing, not only suffered from the highest *C_year_* at 74.64 μg/m^3^, but also experienced a high *MI_ep_* at 88.43 μg/m^3^, *DI_ep_* at 21.33 μg/(m^3^·d), and *AD_ep_* at 0.87. Thus, it was a typical city with severe pollution that would easily accumulate. The reason for that might be related to the high humidity, local multiple emissions, and unfavorable diffusion here [13,20,41]. PM_2.5_ was also easily accumulated in Nantong, Kunshan, Suzhou, as well as Taicang. However, it was low or hard to accumulate in some cities, such as Zhoushan and Chun’an. Among the remaining cities, the PM_2.5_ accumulation degree was at a moderate level.

**Figure 8:**
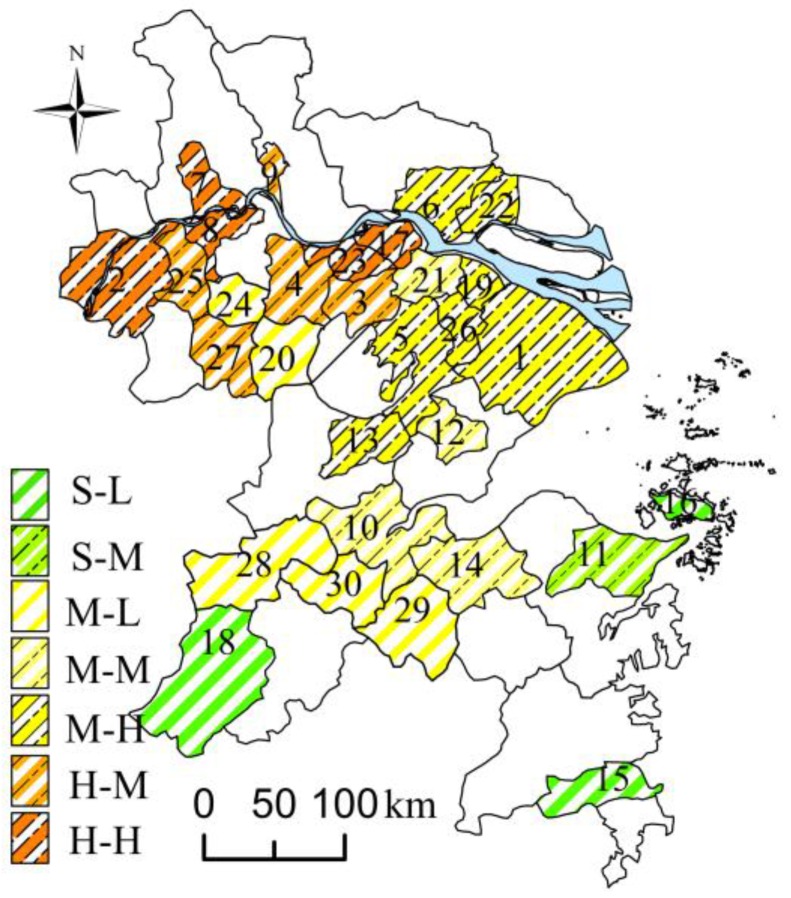
Pollution types of the sample cities. (In this figure, the H, M, and S before ”-” refer heavy, moderate and slight pollution, respectively; the H, M, and L after ”-” refer high, moderate, and low accumulation degree, respectively. Each city was numbered as Figure 1. The blue color is the Yangtze River.)

Based on the equal interval classification method, let the *C_year_* of 30–47, 48–65 and 66–74 μg/m^3^ represent the slight, moderate and heavy pollution level, respectively, and let the *AD_ep_* of 0–0.33, 0.34–0.67 and 0.67–1 refer to the lower, moderate and higher accumulating degree, respectively. A figure indicating PM_2.5_ pollution type was prepared (Figure 8).

Overall, a more heavily polluted city had a higher degree of accumulation of PM_2.5_, although, a less polluted city did not necessarily indicate “lack of ease of accumulation”. In other words, severe pollution was dominated by the ease of PM_2.5_ accumulation in certain spaces. However, in the cities with low *C_year_*, like Shanghai, there were still highly-polluted periods. During the pollution episodes, the increase and decrease of PM_2.5_ were rapid here.

In a word, the difference of the PM_2.5_ accumulation, as well as that of *C_year_*, is significant in the YRD. Unlike the trend of *C_year_*, “higher in the northwest, lower in the southeast”, the tendency of the degree of PM_2.5_ accumulation is “higher in the north, lower in the south”.

### 3.3. Impacts of Socioeconomic Factors on PM_2.5_ Pollution

#### 3.3.1. Pearson Correlation Analysis

Table 3 shows the Pearson correlation coefficients of SEFs with PM_2.5_ episode indexes. From Table 3, it can be seen that no SEF was significantly related to the *C_year_* (R ranging from 0.13 to 0.34), indicating weak correlations between them. However, for PMAE, the coefficients increased remarkably and presented a significant positive relationship. Taken together, three sources, including industrial factors, energy consumption, and population and city, had a stronger influence on PMAE than transportation and agricultural factors. Specifically, nine out of twelve SEFs except for road (X_42_) and agricultural factors (X_51_ and X_52_) were significantly related to *MI_ep_*, as well as to *DI_ep_* and to *AD_ep_* at the level of *p* < 0.01 or *p* < 0.05. Among these nine indicators, firstly, population density (X_32_) had the highest coefficients with *MI_ep_* (0.68), *DI_ep_* (0.67) and *AD_ep_*(0.64), respectively, suggesting that population density could be an important factor driving the rapid increase of PM_2.5_, compared to other factors, when pollution occurs. Although the impact of population density was unclear in previous literature, our results suggested that a higher density of population may influence PM_2.5_ and cause it to increase rapidly, within a short period. Secondly, secondary industry (X_11_), industry above designated size (X_12_), energy consumption (X_21_), total electricity power (X_22_), and electricity power of industry consumption (X_23_) had a high coefficient of 0.53–0.56 with *MI_ep_*, 0.60–0.62 with *DI_ep_* and 0.55–0.58 with *AD_ep_*, indicating these five factors also influence PM_2.5_ accumulation significantly. Overall, compared with *C_year_*, PMEA was more sensitive to the influence of socioeconomic indicators.

#### 3.3.2. *PD* of SEFs to PMAE

The Factor Detector module was used to quantify the *PD* of SEFs to PMAE. The specific process was listed as follows: (1) the SEFs were discretized into five categories (Figure 3); (2) the spatial figures of SEFs were overlaid on the figures of episode indexes (Figure 7) in ArcGIS; and (3) the *PD* of each factor was calculated by the Equation (4). It should be noted we have compared four discretization methods, system cluster, equal interval break, quantile break, and natural break. *PD* values by the system cluster method were larger than the others, thus, we considered it be suitable for the actuality. Furthermore, we also compared four clusters and five clusters and discovered no significant difference between them. Accordingly, this paper chose the system cluster method to discretize the quantitative data into five clusters (Figure 3).

As shown in Figure 3, the twelve SEFs experienced a great difference in the 30 sample cities. The values of CA ranged from 64–167, which indicated that the social and economic gap in the YRD did exist. Generally speaking, the region had an average population of 2.64 million, secondary industry of 1257 billion yuan, urban built-up area of 222 km^2^, and vehicles of 44 million in 2014. Driven by a population of 24 million in Shanghai, most of other SEFs, such as secondary industry, built-up area, vehicles, and energy consumption etc., were more than 100 times higher than that of Chun’an, a city with the minimum of SEFs. In spatial distribution, cities, with a high value of secondary industry, energy consumption, population and population density, were apparently concentrated on both sides of the Yangtze River.

According to Table 3, the *PD* of SEFs to *C_year_* was between 0.09–0.19, while it was in the range of 0.18–0.37 for PMAE, showing an extraordinary increase. Similar to the Pearson correlation analysis, the Factor Detector reached the conclusion that SEFs had a larger explanatory power with regard to the spatial diversity of PMAE than *C_year_*. For *MI_ep_*, population density had the highest *PD* value (0.36), followed by the built-up area (0.33) and population (0.32), suggesting that city size and the population degree could better explain the spatial difference of PM_2.5_ increment compared to the other SEFs. That also means PM_2.5_ accumulates more easily in a larger and more populous city. Similarly, for *DI_ep_*, the high *PD* value belonging to secondary industry (0.37), industry above designated size (0.31), energy consumption (0.32), population (0.30) and population density (0.33), was higher than the other detector factors. Apparently, industry and energy consumption had a very significant influence on the rate of PM_2.5_ increase, as well as population. Synthesizing this information to *AD_ep_*, the *PD* values were sequenced as: X_32_ (0.39) > X_11_ (0.36) > X_23_ (0.34) > X_31_ (0.33) > X_1__2_ (0.32) > X_21_ (0.31) = X_33_ (0.31) > X_41_ (0.23) > X_42_ (0.22) = X_52_ (0.22) > X_22_ (0.19) > X_51_ (0.18). The sequence means that PM_2.5_ was more sensitive to industry factors, energy consumption, population and city size than to transportation and agricultural factors.

**Figure 9:**
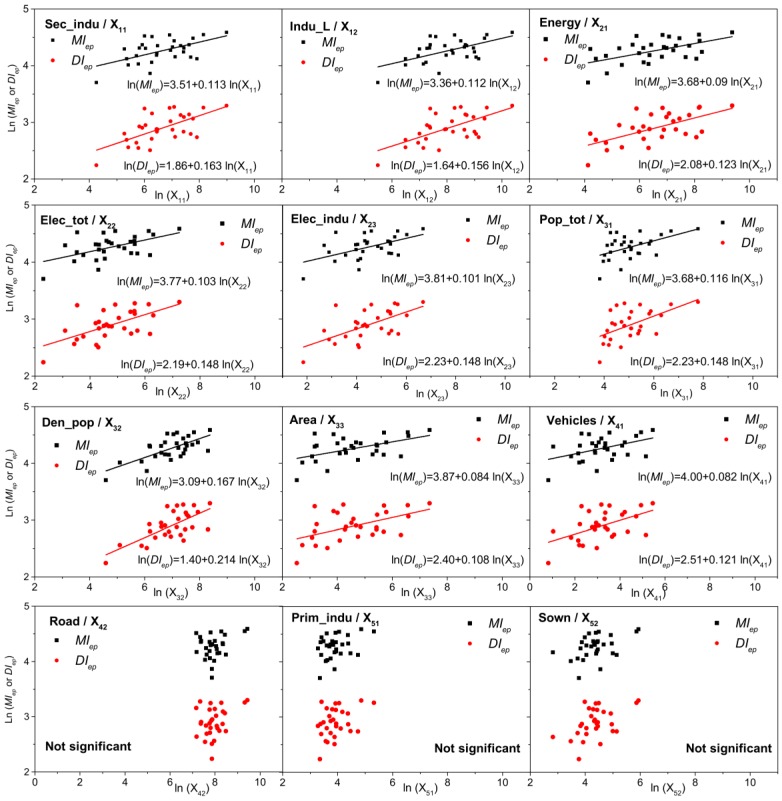
The scatterplots of SEFs with *MI_ep_* (black squares), and with *DI_ep_* (red circles). (Linear fitting between two variables without significant relation was not exerted in this figure.)

The close correlations between PMAE and industrial factors, population and city, and energy consumption could also be observed clearly in Figure 9. Overall, the two upward-sloping fitting lines in each figure (except the last three) mean that a positive interrelationship exists between them. Similar to the Pearson analysis and Factor Detector, population density had the highest slope among the twelve factors, suggesting that a higher increment and rising rate occurred with an increase in population density compared to the other factors. The slopes, 0.17 for *MI_ep_*, and 0.214 for *DI_ep_* showed that an increment of 1% in population density would cause an increase of 0.17% in *MI_ep_* and 0.21% in *DI_ep_.* The other major slopes belonged to industry factors, energy consumption, and vehicles, in that order, showing the importance of these SEFs to PM_2.5_ response during the episodes. From Figure 9, it can be seen that there were no significant linear trends for episode indexes with road and with agricultural factors.

In short, our statistical results indicated that, firstly, PMAE could better explain the distribution of socioeconomic forces on PM_2.5_ compared with the annual average concentration in the YRD on the basis of statistical analysis. Secondly, the population density and total population played important roles in promoting the accumulation of PM_2.5_ during a pollution episode and causing the spatial diversity in the YRD. Thirdly, industrial factors and energy consumption also led to the dramatic increase in PM_2.5_, especially the daily rise rate. Fourthly, as a reflection of the traffic factor, PMAE was significantly related to vehicles, suggesting that the impact of vehicles cannot be ignored. Lastly, although straw burning and agricultural biomass burning have been verified to be a key source in the YRD, we found that the effect of agricultural factors on PMAE was not statistically significant.

## 4. Discussion

During the last few decades, a growing number of scientific papers and reports have explored the socioeconomic drivers of air pollutant emissions, which are important for the development of pollution control strategies. As one of these cases, to better express the socioeconomic impact on haze pollution, we chose to focus only on PM_2.5_ in accumulation stage (PMAE) in this study, unlike previous studies focusing on *C_year_*. The reason was that we considered it may be more reasonable to employ PMAE to reflect the spatial differentiation of socioeconomic impact than *C_year_*.

The following two inferences can be drawn. On the one hand, PM_2.5_ was significantly affected by natural factors such as climate, topography, and surface vegetation [42,43,44] besides socio-economic drivers. *C_year_* is a combined effect of these factors. Particularly, the reduction of PM_2.5_ concentration often depended on precipitation or gales. It is very difficult to identify the influence of each factor on *C_year_*. On the other hand, firstly, the YRD has similar climate conditions. In 2014, the annual average temperature in the 30 cities was between 14–19 °C and the yearly relative humidity was from 71% to 76%. Compared with SEFs in the region, the CA of temperature (6%) and relative humidity (3%) had a sharply decrease, which indicated the difference in weather factors among cities was much smaller than the SEFs. Secondly, one pollution episode only lasts for several days. In a few days, especially during the accumulation phase of a pollution episode, the synoptic conditions are relatively stagnant, which is one of the prerequisites of PM_2.5_ pollution occurrence. Lastly, the ten pollution episodes were strictly picked out for this study under the principle without the interference of inclement weather (rainfall, winds, etc.). Thus, we believe that it can reduce the interference from natural factors as far as possible to focus on PMAE at the regional scale.

Even so, the disturbance caused by the difference in synoptic conditions in the YRD could not be removed completely. In addition, although we realize that the significant distinction of topography and landscape structure in the study area also contributed to the spatial variation of PMAE, this paper had to exclude these factors due to the lack of data. Furthermore, we selected the urban district as the sample assessment unit to minimize the coverage of PM_2.5_, while it is still a limitation that using the data from a small number of fixed monitoring stations to represent the PM_2.5_ pollution of a large area. With reference to existing, similar papers on the relationship of SEFs with PM_2.5_, many articles also explored the relationship between socioeconomic factors and monitoring-site PM_2.5_ [16,29], in which the influence of natural factors are rarely included [5,6]. Further, the data in the logarithmic form in our study could efficiently minimize the potential heteroscedasticity. Therefore, we considered that the exclusion of the influence of natural factors may not result in a serious evaluation bias.

On basis of this, our results showed that SEFs were more closely related to the episode indexes than to the *C_year_*. In view of socioeconomic factors, firstly, although there has been no conclusive result about the positive or negative effects of population density on PM_2.5_ from the existing articles so far, our statistical results indicated that population and its density have a significant influence on PMAE in the sample region. This may be because the population is the leading factor driving the increase in other social and economic factors. For example, in cities with a higher population density, more energy consumption, more private vehicles, and more industrial activities are required to satisfy the needs of a considerable population, which will, in turn, generate more emissions and deteriorate the air quality. When fine particles accumulate to a certain extent under stagnant synoptic conditions, PM_2.5_ pollution will occur or be aggravated. According to existing research [6,38], the conclusion that PM_2.5_ concentration was higher in urban areas than in rural regions also indicated population and its density may cause urban air quality deteriorating. Secondly, indicators of industrial factors, such as secondary industry, industry above designated size and energy consumption showed an important impact on PMAE in our study. In fact, industry, especially heavy industry, and its energy consumption could discharge many pollutants. As Zhao et al. [45] pointed out, a 1% increase in industrial added value will result in an increase of nearly 0.847% in relative pollution density. Therefore, the difference in industrial activities was also responsible for the spatial diversity of PMAE in the YRD. Thirdly, the number of motor vehicles, private vehicles in particular, has increased dramatically in recent years. As many studies have suggested, vehicular emissions include the main components of PM_2.5_, such as particles or the precursor gases [46,47,48]. Through this paper, we also found the significant impact from vehicles on PMAE, based on statistical information. Lastly, the influence of agricultural factors was deemed as a vital source of PM_2.5_ in previous studies [49,50]. However, it was not found to be strong enough to be statistically significant in our study. We inferred that the comparative stability of the agricultural effect could not lead to the extreme increase in PM_2.5_, except in the harvest season, a few episodes during which time were appropriate for our study.

In brief, socio-economic factors heavily influenced the PMAE, with all contributors being tied to each other with the abovementioned analysis. There were two main contributions in this study. Firstly, we proved that the socio-economic influence on pollution episodes was more significant compared to the annual pollution level, which is rarely mentioned in previous studies. Secondly, the influence of each factor on PM_2.5_ episodes was explored by the suitable techniques, geographical detector and linear regression. All of this information would be useful for developing policies for the improvement of air quality, especially during periods of serious haze episodes.

## 5. Conclusions

Using the PM_2.5_ concentrations and twelve socioeconomic indicators in 2014, we have explored, for the first time, the characteristics of PM_2.5_ accumulation during pollution episodes and its links with socioeconomic factors in the YRD from a statistical perspective. To compare, we employed the *C_year_* as the annual average pollution level and defined the episode indexes, *MI_ep_*, *DI_ep_* and *AD_ep_* to represent the degree of accumulation of PM_2.5_ (the average of ten adopted episodes). In general, the spatial pattern of *C_year_* indicated “higher in the northwest, lower in the southeast”, while the high value of PM_2.5_ accumulation degree was mainly distributed among the northern cities located along the Yangtze River. Pearson coefficients and *PD* values suggested a similar conclusion, that the variation in PM_2.5_ during the episodes was more sensitive to socioeconomic impact than *C_year_*. Scatter plots and linear regression further verified the influence of population density in causing PMAE variation in the YRD.

Overall, larger and more populous cities will generate more emissions, with PM_2.5_ accumulation being easier there compared with the smaller ones. It may be time to think about controlling the scale of city expansion and population density in urban planning. Of course, China should also accelerate industrial restructuring, reduce coal consumption and develop new, cleaner energy sources. At the same time, motor vehicles did have a significant effect on PM_2.5_ accumulation, and hence, should not be taken lightly. Although the explanatory power of agriculture was relatively weak in this study, the particles contributed by biomass burning identified by many other studies should be strictly controlled. Furthermore, air pollution was driven by all social and economic activities and their interaction. More studies about understanding the potential mechanism of socio-economic factors need to be conducted for improving the quality of our environment in the future.

## Figures and Tables

**Table 1:** Indicators of potentially influential factors.

Abbreviation	Interpretation of Indicators (Unit)		Max	Min	Mean
***Industrial factors***					
Secondary industry/X_11_	The secondary industry DGP (100 million yuan)		8167	71	1258
Industry above size/X_12_	Gross industry output value above designated size (100 million yuan)		32,665	241	5098
***Energy consumption***					
Energy consumption/X_21_	Total energy consumption (10,000 ton standard coal)		11,084	62	1494
Electricity power/X_22_	Total electricity power (100 million kWh)		1367	10	205
Industry electricity/X_23_	Industry consumption of electricity power (100 million kwh)		785	6	145
***Population and City***					
Population/X_31_	Permanent Resident Population (10,000 persons)		2425	46	264
Population Density/X_32_	Density of Population(person/km^2^)		4341	98	1399
Built-up area/X_33_	Area of urban Built-up (km^2^)		1563	13	216
***Transportation***					
Vehicles/X_41_	Possession of Civil Vehicles (10,000 vehicles)		304	2	44
Road/X_42_	Total length of roads (km)		17,797	1296	3353
***Agricultural factors***					
Primary industry/X_51_	The primary industry DGP (100 million yuan)		205	27	58
Sown/X_52_	Total sown area of crops (1000 hm^2^)		358	17	98

**Table 2:** Descriptive statistics of accumulation indicators during ten episodes in the YRD.

Episode	Month	*MI_ep_*			*DI_ep_*			*AD_ep_*		
		Min	Max	Mean	Min	Max	Mean	Min	Max	Mean
EP1	January	14	167	85	4.67	42.50	23.53	0.00	0.96	0.48
EP2	January	80	217	144	21.09	55.42	32.92	0.06	0.98	0.40
EP3	February	29	107	59	9.67	42.85	18.44	0.00	0.86	0.33
EP4	March	36	131	83	9.37	40.04	22.57	0.01	0.94	0.46
EP5	May	31	187	73	7.01	37.49	15.37	0.01	1.00	0.27
EP6	October	43	129	79	9.73	43.09	20.77	0.02	1.00	0.37
EP7	November	2	117	61	0.70	25.25	15.09	0.01	0.96	0.55
EP8	November	3	56	24	1.43	28.00	10.46	0.00	1.00	0.36
EP9	December	13	98	50	2.60	24.50	12.32	0.00	1.00	0.44
EP10	December	2	141	69	0.78	46.94	22.07	0.00	1.00	0.47
Average	annual	54	90	72	13.46	25.67	19.38	0.00	0.96	0.26

**Table 3:** Correlations between SEFs and PM_2.5_.

Pollution Sources	Detector Indicator	Pearson Coefficients					*PD*		
		*C_year_*	*MI_ep_*	*DI_ep_*	*AD_ep_*	*C_year_*	*MI_ep_*	*DI_ep_*	*AD_ep_*
*Industry factors*	Sec_indu/X_11_	0.18	0.56 **	0.62 **	0.58 **	0.13	0.27	0.37	0.36
	Indu_L/X_12_	0.34	0.54 **	0.61 **	0.55 **	0.19	0.26	0.31	0.32
*Energy consumption*	Energy/X_21_	0.19	0.55 **	0.61 **	0.58 **	0.12	0.30	0.32	0.31
	Elec_tot/X_21_	0.20	0.53 **	0.60 **	0.55 **	0.17	0.18	0.18	0.19
	Elec_indu/X_23_	0.33	0.53 **	0.62 **	0.57 **	0.15	0.29	0.31	0.34
*Population and city*	Pop_tot/X_31_	0.22	0.46 *	0.56 **	0.54 **	0.13	0.31	0.29	0.33
	Den_pop/X_32_	0.22	0.68 **	0.67 **	0.65 **	0.12	0.36	0.33	*0.39*
	Area/X_33_	0.13	0.47 **	0.48 **	0.47 **	0.11	0.33	0.29	0.31
*Transportation*	Vehicles/X_41_	0.21	0.43 *	0.50 **	0.46 *	0.10	0.19	0.20	0.23
	Road/X_42_	0.23	0.32	0.31	0.31	0.09	0.18	0.19	0.22
*Agriculture factors*	Prim_indu/X_51_	0.15	0.25	0.29	0.27	0.12	0.21	0.14	0.18
	Sown/X_51_	0.34	0.36	0.37	0.36	0.18	0.23	0.17	0.22

** *p* < 0.01, * *p* < 0.05.
